# Nurses’ and occupational therapists’ experiences of conducting a home-based psychosocial intervention following stroke: a qualitative process evaluation

**DOI:** 10.1186/s12913-021-06857-8

**Published:** 2021-08-11

**Authors:** Randi Martinsen, Gabriele Kitzmüller, Margrete Mangset, Kari Kvigne, Anne Svelstad Evju, Berit Arnesveen Bronken, Line Kildal Bragstad, Ellen Gabrielsen Hjelle, Unni Sveen, Marit Kirkevold

**Affiliations:** 1 Department of Health and Nursing Sciences, Faculty of Social and Health Sciences, Inland Norway University of Applied Sciences, P.B. 400, 2418 Elverum, Norway; 2 Department of Health and Care Sciences, Faculty of Health Sciences, UIT The Arctic University of Norway, Tromsø, Norway; 3 Department of Geriatric Medicine, Oslo University Hospital, Oslo, Norway; 4 Department of Nursing Science and Research Center for habilitation and rehabilitation services and models (CHARM), Faculty of Medicine, University of Oslo, Oslo, Norway; 5 Department of Geriatric Medicine and Physical Medicine and Rehabilitation, Oslo University Hospital, Oslo, Norway

**Keywords:** Dialogues, Experiences, Focus groups, Health care professionals, Intervention, Process evaluation, Psychosocial, Stroke, Qualitative

## Abstract

**Background:**

Persons with stroke are susceptible to psychosocial problems, and express disappointment at how health care professionals fail to meet their psychosocial needs following discharge to home. The responsibility of nurses and occupational therapists in stroke rehabilitation is to assist the persons and their families during the recovery and adjustment process. A home-based dialogical intervention aiming to enhance psychosocial support was therefore developed and tested in a randomized controlled trial. This study is a part of the process evaluation conducted alongside the trial. The aim was to explore the nurses’ and occupational therapists’ experiences of conducting the intervention.

**Methods:**

Eighteen nurses and four occupational therapists participated in six focus groups to explore their experiences when providing the intervention. The themes discussed in the focus groups were the aspects that facilitated the delivering of the intervention and the challenges they encountered during the study period. The interviews were analysed using qualitative content analysis.

**Results:**

The analysis generated two themes. The theme Developing a supportive relationship to facilitate the adjustment process following stroke had two subthemes: *Getting personally involved* and *Handling challenges*. This theme reveals how the nurses and occupational therapists experienced their relationship with the persons with stroke and potential threats which challenged them while conducting the intervention. The theme Developing professional skills in providing psychosocial support had two subthemes: *Becoming confident in conducting dialogues* and *Integrating psychosocial topics*. This theme reveals the aspects that the nurses and occupational therapists perceived as facilitating the development of their professional skills in conducting the dialogues.

**Conclusion:**

Delivering the psychosocial intervention was perceived as deeply meaningful and increased the nurses’ and occupational therapists’ understanding of how to support stroke survivors to live with the consequences of stroke. However, balancing the professional and the personal relationship was challenging. A basic educational programme, training, supervision and having dedicated time were crucial elements to instil confidence in professionals conducting theme-based dialogues to promote post-stroke psychosocial well-being. Individual clinical experience and knowledge of stroke care were considering important to enable professionals to integrate psychosocial rehabilitation into community health care.

**Trial registration:**

ClinicalTrials.gov, NCT 02338869, registered 10/04/2014.

## Background

The responsibility of health care professionals in stroke rehabilitation is to assist the persons and their families during the recovery and adjustment process according to stroke rehabilitation guidelines [1]. Psychosocial support should address the person’s various challenges and needs after stroke. Both information and supportive dialogues are needed to help the person to adjust to an altered life situation and develop new life skills [2].

The psychosocial challenges following a stroke are known to influence the recovery process of the individual as well as making it difficult to maintain family life, employment and social networks [3–6]. Anxiety, depressive symptoms, social isolation, fatigue, aphasia and cognitive decline are well documented risk factors for well-being [7–9] and affect the possibility of living a meaningful life following a stroke [10]. The adjustment process involves psychosocial problems which need to be addressed after discharge [10–13].

The importance of addressing post-stroke psychosocial needs is emphasized [13–15]. Persons with stroke themselves miss integrated strategies to prevent psychosocial problems [10, 11, 16–18] and express disappointment at how health care professionals meet their individual needs and expectations during the rehabilitation process [5, 11, 13, 16, 19]. However, even though health care personnel are aware of the importance of addressing psychosocial needs during rehabilitation, there is still little evidence that these needs are systematically addressed in stroke rehabilitation [13, 15].

Insufficient professional competence seems to be a general barrier to achieve change in community health care [20]. Shortage of time is also known to interfere with the ability to provide support during everyday health care in the community [20–22].

Due to better diagnostic procedures and shorter hospital stays, many persons with stroke are now discharged earlier than before [10, 23]. The transition to home means facing new challenges during the recovery and adjustment process [12] which increases the need for professional psychosocial support [13]. Therefore, a dialogue-based intervention aiming to enhance psychosocial support was developed [24, 25] and tested in a randomized controlled trial (RCT) [26, 27].

In line with the Medical Research Council framework for developing complex interventions [28], process evaluation alongside the clinical trial is recommended to deepen the understanding of the intervention processes and strengthen the overall quality of the RCT. The process evaluation includes an in-depth exploration of the intervention personnel’s experiences of conducting the intervention, their impression of potential threats and facilitators for delivering the intervention.

The specific aim of the current study was to explore the experiences of nurses (RNs) and occupational therapists (OTs) of conducting a psychosocial home-based intervention with persons with stroke during the RCT.

The following research questions were formulated:

- Which aspects did the RNs and OTs perceive as important for delivering the intervention to the persons with stroke?
- What challenges did the RNs and OTs encounter when delivering the intervention?

## Material and methods

### The study design

This study had a qualitative design using focus groups to collect data. The study was a part of the process evaluation conducted alongside the multicentre randomized controlled trial ‘Psychosocial well-being following stroke’ conducted in Norway.

This study has been reported in accordance with the consolidated criteria for reporting qualitative research (COREQ) [29].

### The intervention

In their regular roles, both RNs and OTs are working to support coping and adjustment processes after stroke. Thus, both professionals were perceived to have sufficient knowledge and professional competencies to conduct the intervention dialogues. In the intervention, the RNs and OTs (i.e. intervention personnel) provided psychosocial support in eight individualized and theme-based dialogues of about 1 h each during a five-month period starting 4 weeks after the stroke. The intervention personnel (IP) provided support and guidance to the persons with stroke in home settings and encouraged them to share and reflect on their thoughts and feelings about their psychosocial situation. Additionally, the dialogues sought to empower the persons to be in charge of their own recovery and adjustment process and to develop new life skills [30].

The IP were certified following a three-day interactive educational programme consisting of lectures, practical training, group reflections and discussions. A manual describing the aim and content of the eight dialogues accompanied by work sheets with relevant stroke-related topics (bodily changes, emotional challenges, significant relationships and social network, daily life issues, meaningful activities, existential issues, self-esteem and identity and problem solving in daily life) supported the IP when conducting the intervention [24, 30].

The IP participated in supervision groups led by members of the research team throughout the intervention period and were invited to contact the research team for advice between the sessions if necessary [30].

For further description of the intervention, see the protocol article [30].

### Participants and recruitment

All the twenty-seven IP who had completed the educational programme and conducted at least one intervention were invited by e-mail to participate*.* Five declined to participate due to a heavy workload, change of residence or other reasons. Finally, twenty-one women and one man gave their informed consent to participate in the study. Five participants had delivered the dialogue-based interventions six to 18 months post-stroke in a parallel study outside the RCT. These five IP were also certified by the research team after completing the same educational programme. They were included because they had valuable experiences with delivering the intervention to persons with stroke later in their rehabilitation trajectory.

Characteristics of the IP are presented in Table 1.

**Table 1:** *Characteristics of the intervention personnel*

	Median (range)
**Age (years)**	53.5 (31–65)
**Work experience (years)**	
Work experience since professional education	20.5 (8–40)
Work experience with stroke patients	10.0 (2–24)
Number of interventions per professional	3 (1–8)
	***N*(%)**
**Professional background**	
Registered nurse	18 (81.8)
Occupational therapist	4 (18.2)
**Education**	
Master’s degree	6 (27.3)
Further education after bachelor’s degree	9 (40.9)
Bachelor’s degree	7 (31.8)

### Data collection

Focus groups were conducted to explore the IP’s experiences of carrying out the dialogue-based intervention, with the aim of eliciting rich data through enabling discussions and interaction between the IP in the group [31]. Focus groups are useful to provide experiences of a topic and the method has been used frequently to evaluate interventions [32].

All members of the research team were involved in the development of the interview guide and four members of the research team led the focus groups.

Initially, four focus groups with five to six participants were arranged. However, some of the participants were not able to participate within the planned time schedule for the focus groups. For that reason, one group was conducted with two participants. Two of the other groups had three participants, one group had four participants, while the last two groups had five participants. We deemed that all the IP experiences were valuable, and all focus groups covered rich discussions related to the topics in the interview guide. All focus groups were conducted in the period from December 2016 to July 2017. Each group was led by two members of the research team in accordance with recommendations for conducting focus groups [31–33]. The moderator acted as a facilitator to elicit the focus group members’ collective perspective on their experiences. The assistant moderator acted as an observer and made sure that the interview themes had been elucidated in detail before finishing each interview [32]. The interview guide was structured thematically opening with ‘Could you please tell us about your experiences of conducting the intervention?’ The moderator made sure to include all participants, encourage discussions, clarify positions and probe for deeper responses [32]. The task of the moderator was also to dwell on important themes related to the topics in the interview guide which included participants’ experiences when providing the intervention, the development of their professional skills during the intervention, the influence of their professional background and the importance of the educational programme for their ability to provide the intervention according to the protocol.

We aimed to establish an open and trusting atmosphere during the interviews to encourage the participants to share experiences of success and failure.

The focus groups lasted between 87 and 104 min and were audiotaped and transcribed verbatim.

### Analysis

Qualitative content analysis with a hermeneutical approach was used to explore the manifest and latent content of the text [34]. Graneheim and Lundman’s approach [34] guided the analysis. An inductive approach was employed where the text was searched for similarities and differences in the data [34–36]. Initially, six members of the research team (i.e. the core group) read the six interview texts individually to gain an overall sense of the IP’s experiences of delivering the intervention, which aspects they perceived as important and the challenges they faced during the intervention. Thereafter, the core group defined and condensed the meaning units and performed the initial coding. The main issues of the coding process related to the educational programme, conducting the dialogues, relationships with the persons with stroke, lessons learned, and challenges of delivering the intervention. Following the initial coding, the texts were discussed in the core group and a revised coding scheme was developed based on the condensed meaning units. Each member of the core group coded the texts using the revised coding scheme. Interrelated codes were organized into categories and subcategories. The manifest content revealed that all participants in the six focus groups agreed upon the issues covered in the discussions.. To arrive at the latent content of the analysis, interpretations of possible underlying meanings were discussed in the core group and the research group. For examples of the analysis process, see Table 2. Finally, the research group members reached consensus on the following four subthemes: *Getting personally involved, Handling challenges, Becoming confident in conducting dialogues and Integrating psychosocial topics.* At the end, the results were abstracted to the two following themes Developing a supportive relationship to facilitate the adjustment process following stroke and Developing professional skills in providing psychosocial support.

**Table 2:** Examples of the analysis process and overview of the themes and subthemes

Meaning units	Codes	Categories	Subthemes	Themes
‘I feel enriched that I’ve been able to come and meet them at home. She’s shown me so much trust and we had a very good dialogue. It’s felt very special and I looked forward to our meetings’.	Engagement	Establishing a relationship	*Getting personally involved*	Developing a supportive relationship to facilitate the adjustment process following stroke
‘You go to their homes and get to know them. You try to stop, but they get attached to you, you’re a person who’s been important in part of their life. One of them asked if we could be friends on Facebook. I found that difficult – without rejecting her. They can easily see you as more private than you are’.	Relationship	Interaction over time	*Handling challenges*	
‘[I] feel I’ve gained even more experience in how to ask, ask the right questions to get a good dialogue, or get the patient to think’.	Precondition	Precondition for performing a role	*Becoming confident in conducting dialogues*	Developing professional skills in providing psychosocial support
‘I thought of Antonovsky’s theory about a meaningful context, that it’s important to belong somewhere and feel a sense of coherence. There was one patient who said he saw no point in living and then I thought about health promotion and how I could find his resources’.	Foundation	Theoretical foundation	*Integrating psychosocial topics*	

## Results

The focus groups revealed that the RNs and OTs welcomed the opportunity to provide psychosocial support through individual theme-based dialogues in the patients’ home environments. Having dedicated time for conducting the dialogues during the intervention study motivated the IP, was experienced as valuable and enhanced the possibility to dwell upon the patients’ psychosocial needs in line with the topics in the dialogues and the intervention protocol.

The IP highlighted their own clinical competencies and experiences in stroke nursing or occupational therapy as key aspects in their ability to evaluate and address patients’ psychosocial problems to facilitate their psychosocial rehabilitation process.

The results are further described in the two themes Developing a supportive relationship to facilitate the adjustment process following stroke and Developing professional skills in providing psychosocial support.

### Developing a supportive relationship to facilitate the adjustment process following stroke

This theme reveals how the IP experienced the relationship with the persons with stroke during the study period and potential threats which challenged them when conducting the intervention, presented in the subthemes *Getting personally involved* and *Handling challenges*.

#### Getting personally involved

All IP stated that the repeated sessions in the patients’ homes enabled the establishment of a trusting relationship and personal involvement in the patients’ lives. The relationship that gradually developed between the stroke survivor and the IP during the eight sessions was perceived as a meaningful experience. They found that the patients looked forward to their visits. Following the patients over a five-month period expanded the IP’s insight into the patients’ lived lives, which was difficult to achieve in their usual work.

*I feel enriched that I’ve been able to come and meet them at home. She’s shown me so much trust and we had a very good dialogue. It’s felt very special and I looked forward to our meetings.* (RN)

Patients had a wide variety of personal needs and illness narratives. Listening to their experiences of bodily changes and descriptions of changes in their lives and relationships enhanced the IP’s understanding of the multiple individual needs following stroke. Thus, the IP needed to open up and to be receptive and engaged during the dialogues and to individualize every dialogue session, as seen in the following quote:

*She was very open and honest and talked about relationships she found difficult in her social network. I think we achieved a good balance in our relationship, but I needed to reflect on it.* (OT)

Another IP expressed her experience in this way:

*She was an 87-year-old lady, so then I had to spend more time to help her feel secure with me. But eventually it got to be really nice.* (RN)

The intervention included different themes that enabled the IP to respond emotionally and enhanced their focus on existential issues.

*It’s hard to see people struggling. She thought it was awful, she was crying and she felt trapped. It* [was] *so important and terrible for her. That does something to you. You leave with a slightly different feeling than when you arrived.* (RN)

*These* [are] *very existential things you experience, like the question of life and death. I was very touched by meeting her.* (OT)

Some IP were surprised at their own engagement in the lives of the persons with stroke and at how much they were thinking of them between the encounters.

*My level of engagement surprised me: I was surprised at the amount of thoughts I invested in the stroke survivor between the meetings.* (RN)

The dialogues enhanced the IP’s understanding of complex dynamics of family relationships and influences of family roles following a stroke, such as a difficult marriage, challenges in trying to return to work or having one’s driving licence suspended.

*It was a real eye-opener for me to* [be so close to] *another person’s life* [and see] *how it* [the stroke] *changes previous roles. That was a powerful experience.* (RN)

The dialogues also focused on other topics unrelated to stroke, but still affecting the life of the patient.

*The topic of conversation wasn’t only stroke, but also other aspects of life, such as having a partner with dementia, past experiences of grief, and practical things*. (RN)

The theme ‘getting personally involved’ demonstrates that delivering the intervention was perceived as deeply meaningful and increased the IP’s understanding of living with the consequences of the stroke. To understand how the consequences of stroke affect the life of the individual and the family seems to be a premise for providing person-centred psychosocial support.

#### Handling challenges

The IP sometimes found it challenging to keep the relationship on a professional level, because the persons with stroke expected that the relationship was developing into a more personal one. Dealing with the increasing familiarity could be demanding, as one of the IP expressed when the person with stroke tried to make contact between the scheduled encounters.

*You go to their homes and get to know them. You try to stop, but they get attached to you, you’re a person who’s been important in part of their life. One of them asked if we could be friends on Facebook. I found that difficult – without rejecting her. They can easily see you as more private than you are.* (RN)

Other challenges the IP discussed during the focus group interviews were when the persons with stroke wanted to give them presents or invite them to dinner. Similarly, situations where the persons with stroke flirted with the IP made them feel uncomfortable.

*I had one* [who] *started to turn on the charm a lot after a few visits. I found that very unpleasant. I wondered whether I’d encouraged it in some way. I don’t think I’d done anything wrong, but I’ve thought about it a lot.* (RN)

Some encounters made the IP reflect on their own values and norms and how those affected their role and expectations for the patients. This was especially the case if the person’s lifestyle differed from the norms of the health care professional and those of society in general.

*I felt I had to do a bit of introspection. It was my inner thinking* [about] *how I should relate to him when I met him. I was a bit unsure at first, but then it worked out ok.* (OT)

Sometimes, practical issues affected the continuity of the intervention and the quality of the relationship between the IP and the person with stroke. Some patients were reluctant to make appointments or find time for the meetings due to their work or other activities. In such cases, some IP felt that they were imposing upon the person or disrupting the person’s life. Some persons with stroke stated that they participated in the project primarily to support the research. In some of these cases, the IP found it challenging to engage the patients in focusing on their psychosocial health. Being unable to engage these participants made the IP question their own professional skills.

Involving family members in some of the dialogues was in accordance with the protocol, but also sometimes necessary for the intervention process, to enable the persons with stroke to express their needs.

*His wife took part too, but not always. He had such a bad memory, so it helped a lot to have her there*. (RN)

The presence of the spouse or another family member could also dominate the dialogues and hinder the patients in expressing their own personal needs, thus making it difficult for the IP to conduct the sessions.

*His wife had a different view of* [the situation] *than* [the person with stroke]*. They viewed his condition differently. He had to tell me how good he was at doing things and everything he could do, but she thought he was pretty useless. Who should you believe and who should you side with?* (RN)

Some IP also found that family members were present during the sessions without being actively involved in the dialogues. Nevertheless, the IP highlighted that being present during the meetings could provide the family member with more information about the ill person’s struggles.

### Developing professional skills in providing psychosocial support

This theme reveals the IP’s experiences of factors that helped them to develop their professional skills in conducing the dialogues, presented in the subthemes *Becoming confident in conducting dialogues* and *Integrating psychosocial topics*.

#### Becoming confident in conducting dialogues

None of the RNs or OTs had previous experience in providing theme-based dialogical support to stroke patients at home. They found that conducting the intervention had developed their professional and personal skills.

[The project was] *very worthwhile for me to be a part of. In terms of my role as a professional there was a lot to be learned.* (RN)

The IP illustrated how the dialogues and guidance were performed. They described how they gradually developed their skills to meet the complexity of needs and how they progressed from feeling unskilled in conducting dialogues towards being confident and supportive in their role of providing advice and guidance.

*In the last session, I felt I’d done this many times. I felt freer* [and I was] *more confident in my role as an interventionist*. (RN)

However, those who did not have the opportunity to engage with more than one person with stroke realized that conducting more dialogues might have enhanced their skills. The manual and work sheets were helpful in preparing for the sessions, reflecting on the topics and maintaining continuity.

The IP stated that they acquired better communication skills and learned how to ask questions that made patients more aware of their own resources. In addition, they learned how to elaborate on important topics.

[I] *feel I’ve gained even more experience in how to ask, ask the right questions to get a good dialogue, or get the patient to think.* (RN)

The IP highlighted how they developed their guidance skills and adjusted them to patients’ individual rehabilitation trajectories. Although most of the IP were experienced in stroke care, they expressed a lack of skill in communicating with people with aphasia. They stated that the educational programme had been important to enhance their understanding of how to provide advice and guidance to patients with conversation problems.

*The real eye-opener for me was the aphasia approach - how to relate to patients with aphasia.* (RN)

#### Integrating psychosocial topics

The IP highlighted how being involved in the intervention enhanced their awareness of health promotion in community stroke care. They exemplified in different ways how they integrated the theoretical framework of the intervention into the dialogues and assisted the persons with stroke in identifying difficulties and strengths, making them conscious of their psychosocial situation and helping them to identify realistic goals, as outlined in the framework.

*I thought of Antonovsky’s theory about a meaningful context, that it’s important to belong somewhere and feel a sense of coherence. There was one patient who said he saw no point in living and then I thought about health promotion and how I could find his resources.* (RN)

To empower the persons with stroke in their struggles to adjust their lives to the consequences of the stroke was a novel way of thinking and working. The IP found that the perspectives of reciprocity, empowerment and well-being differed from their previous experiences of supporting patients. The focus of the intervention on strengthening patients’ ability to share their illness narratives made the IP skilled in addressing the multiple consequences of the stroke. Their focus on dialogue became more important than other aspects they had previously focused on in stroke rehabilitation.

*Having time to address depression, anxiety and the fear of a new stroke and* [having] *time to go into those things properly* [was very important]. *Because people are always going on about exercise and let’s get you up and walking again. There’s a focus on language training or other training. But dialogues can be just as important - or even more* [important]*.* (RN)

Although the IP were experienced in stroke care, all underlined that they found the educational programme important and inspiring.

*The educational seminars were an important foundation for the intervention. I felt they gave me a lot of confidence.* (RN)

The educational programme made them feel prepared and confident to be able to integrate psychosocial issues into clinical practice.

## Discussion

The aim of this study was to explore experiences of RNs and OTs of conducting a dialogue-based intervention for persons with stroke at home. More specifically, we explored the aspects the RNs and OTs found important for delivering the intervention to persons with stroke in their home and the kind of challenges they encountered in the process.

Delivering the psychosocial intervention was perceived as deeply meaningful. The interpersonal interaction during the dialogues enabled the IP to become acquainted with the persons with stroke. The manual and work sheets were also perceived as helpful tools to keep the dialogues focused on the individual’s specific situation. The non-instrumental way of communicating and asking questions about inner thoughts on living with the consequences of a stroke during several meetings made them feel successful and better skilled in providing psychosocial support. Conducting the dialogues in the homes over time facilitated the IP’s awareness of the wide-ranging life situations following a stroke. This is in line with the study by Wottrich et al. [37], who also found the home environment to be a suitable setting to become familiar with individual life stories and to link pre-stroke and post-stroke life. The home environment was also found to be useful for exploring experiences and needs, as well as observing and assessing collaboration with family members [37]. In our study, meeting the persons with stroke in their homes added valuable information about their lives and provided an opportunity to know each individual in depth. To know the individuals in depth was considered important in addressing psychosocial needs, even though the home environment sometimes made it difficult for the IP to keep the relationship on a professional level.

The IP participating in this study expressed dedication to performing the intervention, which they found valuable for the persons with stroke, and their personal involvement over time increased their professional understanding of how it was to live with stroke. This also increased their skills in addressing psychosocial topics. The positive influence of participating in the intervention sessions was also confirmed by most of the persons with stroke included in the in-depth interviews in a linked sub-study within the larger study [38]. Most of them found the dialogues meaningful and felt that the IP listening to their illness stories helped them to reflect upon their new situation and communicate their resources, fears and worries. These findings also concur with some of our previous feasibility studies, which have highlighted patients’ need for continual support and the possibility to talk to a trusted professional about threats to well-being following a stroke [12, 25, 39, 40]. Some also found that participation in the dialogues had a positive impact on their family relationships [38].

The allotted time of eight sessions of about 1 h each was emphasized as helpful in developing the interpersonal relationships. Shortage of time was revealed as a barrier to conduct interventions in the study by Munce et al. [22] and in the systematic review by Lau et al. [20]. Thus, having enough time to conduct dialogues is a prerequisite for developing interpersonal relationships with persons with stroke, a patient group known to struggle with cognitive challenges [9, 41].

Although conducting dialogues and establishing relationships over time was enriching, some situations were also occasionally perceived as demanding. The IP had to engage in each person’s individual and complex adjustment process. Such personal involvement posed a risk of crossing the line between acting like an IP and acting like a friend. This emphasizes the importance of supervision and the opportunity to discuss how to handle complex problems with other professionals. Conducting theme-based dialogues systematically over time to enhance well-being was experienced by the IP as a rather unfamiliar and novel way to communicate for the IPs. A lack of confidence in how to manage demanding situations could in some instances make the IP uncomfortable. This corresponds with the study by Horne et al. [42], who found that professionals who conducted interventions could feel uncomfortable despite having previously received appropriate training. The importance of facilitation to succeed in implementing intervention programmes is discussed by Munce et al. [22]. As demonstrated in the findings, the IP needed both theoretical and practical training. Having an active role over time in interventions enhances learning and improves confidence [43]. A basic educational programme, training and supervision seem to be necessary components to develop confidence and facilitate health personnel in providing psychosocial support. In this study, the IP also assessed their clinical and professional experience as decisive in developing a professional attitude to addressing psychosocial topics during theme-based dialogues. This might suggest that clinical experience as a stroke health care professional is a further prerequisite for confidence in conducting theme-based dialogues in stroke care, in addition to training and supervision.

Not all persons with stroke found the dialogues useful [38], highlighting that not all patients are interested in, or in need of, psychosocial support following a stroke, which is also highlighted by Northcott and colleagues [44]. However, in light of the high rates of psychological distress and depression following stroke [9, 45], there is clearly a need to identify patients who can benefit from psychosocial rehabilitation programmes.

### Methodological considerations

Twenty-two of the twenty-seven IP who conducted the dialogues took part in the focus groups. The high number of participants engaging in the focus groups strengthens the study. In addition, participants varied in age, educational level and professional experience in stroke care, as well as in the number of interventions they conducted. These variations shed light on different experiences in conducting the intervention and provided rich data.

Both RNs and OTs performed the interventions. Differences between the education and everyday work practice of these two professional groups could have influenced how they implemented the intervention and experienced conducting the dialogues. However, any differences were minimized through the joint educational programme, the manual describing the aim and content of the eight dialogues, and the supervision arranged throughout the study period. One of the process evaluation studies showed that the IP conducted the dialogues according to the protocol, demonstrating a high level of protocol adherence in 80% of the interventions [46].

One of the focus groups had only two participants, two consisted of three participants, while the remainder had four or five participants. The small number of participants in some of the focus groups might have influenced the depth of the discussions in different groups and thus the results. However, all focus groups had dynamic and fruitful discussions that revealed different viewpoints. Other studies have demonstrated that a small number might allow all participants to be included in useful discussions, giving more time for the experiences of each person and thus providing rich data [47, 48].

All the members of the research team of three OTs and six RNs were skilled in stroke care and/or stroke research. The intersubjectivity and transparency was strengthened by involving all the researchers in developing the interview guide, in discussions of the manifest and latent content, and in the creation of categories and themes of the analysis, and by maintaining open discussions and reaching consensus on the findings. Quotes from the participants underline examples from the focus group discussions and increase the trustworthiness of the interpretations.

## Conclusion

Delivering the psychosocial intervention was perceived as deeply meaningful and increased the IP’s understanding of living with the consequences of stroke. The RNs and OTs welcomed the opportunity to provide psychosocial support during the intervention sessions. Repeated sessions in patients’ homes and the allotted time were experienced as a potential for becoming personally involved, and providing important, dedicated and successful individual support. The interpersonal relationships and involvement established over time developed their professional skills and facilitated the theme-based dialogues. Involvement in patients’ individual life situations was sometimes experienced as challenging in the attempt to find a balance between professionalism and privacy.

### Clinical implications

A basic educational programme, training, supervision and having dedicated time are crucial elements to instil confidence in professionals conducting theme-based dialogues to enhance post-stroke psychosocial well-being. Individual clinical experience and knowledge of stroke care are additional pivotal elements that are important to enable professionals to integrate psychosocial rehabilitation into community health care. The issues involved in how to conduct theme-based dialogues in this study can provide ideas for designing programmes to support other groups needing psychosocial support in community health care. The study results may also help to improve awareness regarding possible barriers and challenges in managing relational aspects with intervention recipients.

## Data Availability

The transcripts (in Norwegian) are available from the corresponding author on reasonable request.

## Abbreviations

RN: Registered nurse
OT: Occupational therapist
IP: Intervention personnel
